# Type 1 and Type 2 Diabetes Measurement Using Human Face Skin Region

**DOI:** 10.1155/2023/9931010

**Published:** 2023-09-26

**Authors:** L. Aneesh Euprazia, A. Rajeswari, K. K. Thyagharajan, N. R. Shanker

**Affiliations:** ^1^Computer Science and Engineering, Velammal Engineering College, Chennai, India; ^2^Electronics and Communication Engineering, R.M.D Engineering College, Chennai, India; ^3^Department of Computer Science and Engineering, Aalim Muhammed Salegh College of Engineering, Chennai, India

## Abstract

**Aim:**

Analyse the diabetes mellitus (DM) of a person through the facial skin region using vision diabetology. Diabetes mellitus is caused by persistent high blood glucose levels and related complications, which show variation in facial skin regions due to reduced blood flow in the facial arteries. *Materials and Method*. In this study, 200 facial images of diabetes patients with skin conditions such as Bell's palsy, rubeosis faciei, scleroderma, and vitiligo were collected from existing face videos. Moreover, face images are collected from diabetic persons in India. Viola Jones' face-detecting algorithm extracts face skin regions from a diabetic person's face image in video frames. The affected skin area on the diabetic person's face is detected using HSV colour model segmentation. The proposed multiwavelet transform convolutional neural network (MWTCNN) extracts the features for diabetic measurement from up- and downfacial scaled images of diabetic persons.

**Results:**

The existing deep learning models are compared with the proposed MWTCNN model, which provides the highest accuracy of 98.3%.

**Conclusion:**

The facial skin region-based diabetic measurement avoids pricking of the serum and is used for continuous glucose monitoring.

## 1. Introduction

Diabetes mellitus (DM) is a group of illnesses characterised by changes in blood glucose levels. DM affects 382 million adults worldwide [1]. According to the research, pathological lesions in the skin may occur in 30 to 70% of patients with DM [1]. The number of people with diabetes in India was estimated to be 77 million in 2019 and 783 million by 2045 [2]. Type 1 DM is about 5% to 10% of the total diabetic-affected person count and is characterised by the particular autoimmune destruction of insulin-secreting b-cells in the pancreas. Dermatological issues occur in 30% of patients [3]. Infections of the skin are more common in type 1 and type 2 DM. Skin manifestations occur due to DM [4]. Patients with diabetes are impacted regardless of their age or gender, and as the DM progresses, chronic degenerative consequences and acute metabolic disturbances impact the skin.

The frequency and key clinical characteristics of skin problems are high in patients with insulin-dependent (IDDM) or noninsulin-dependent (NIDDM) diabetes [5]. The most common dermatological issues in IDDM and NIDDM patients were vitiligo and psoriasis, cutaneous [6] necrobiosis lipoidica [7], diabetic dermopathy [8], diabetic bullae [9], yellow skin [10], eruptive xanthomas [11], perforating disorders [12], acanthosis nigricans [13], and oral leucoplakia [14].

**Figure 1:**
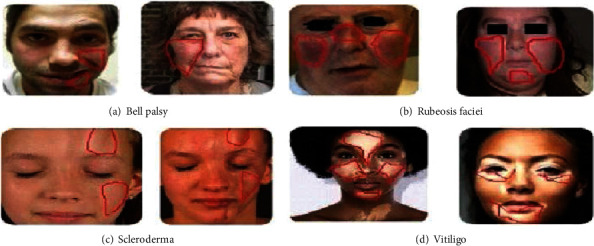
Facial region affected by small blood vessel damage due to type 1 and type2 diabetes [15–19].

According to the American Diabetes Association (ADA) [20], dermatological issues are the first diabetic indication in a diabetic person (Figure 1). High glucose levels harm blood vessels. Circulation and blood flow in the skin are reduced due to damaged blood vessels. The skin's protein structure (collagen) is changed because of decreased blood flow. The changes in collagen affect the healing capacity, texture, and look of the skin. It is frequently a marker of poor glycaemic control. Hyperglycaemia in diabetes causes inactive microcirculation, clinically visible as face vein deformation. Hence, due to DM, vitiligo, rubeosis faciei, Bell's palsy, and scleroderma, skin conditions occur in the face. Vitiligo is simply the loss of skin melanin or skin colour pigment. The internal immunological process can cause the skin to lose colour everywhere, including the face. Vitiligo develops around the eyes, lips, and chin. Vitiligo is an acquired, noncontiguous condition characterised by gradual, patchy skin pigmentation loss, typically covering the hair and mucous membranes caused by melanocyte loss in the affected areas [21, 22]. Vitiligo occurs in both type 1 and type 2 DM patients. Vitiligo occurs around the facial regions such as the eyes, nose, and mouth. The symptoms of vitiligo are milky, white skin. Deep learning and machine learning algorithms are used to detect white skin patches early, such as the global adaptive thresholding algorithm [16, 23] and YOLOV3 [24].

DM manifests cutaneous as rubeosis faciei diabeticorum [17]. It is distinguished by diffuse, persistent facial erythema in diabetes patients. Histologically, diabetic individuals' cheeks have an increase in the number and width of superficial venules and butterfly-shaped cheek redness. The cheek's redness is identified automatically through temporal facial expression [25], histogram [26], and geometric transformation [18]. The most frequent cause of lower motor neuron facial palsy is Bell's palsy (BP) [27], an unpredictable, severe central facial nerve disorder. As a result of cranial nerve VII (the facial nerve), which stimulates the facial muscles, malfunctioning, BP is a sudden weakening or paralysis of the muscles on one side of the face. As a result of an immunological condition or a viral infection, the facial nerve swells in this condition. The nerve becomes compressed, and its blood supply is decreased due to swelling. BP is detected automatically through a key point detection algorithm [28, 29], facial landmark algorithm [30], temperature and texture features [31], and CNN [32]. Diabetes type 1 causes scleroderma [33]. The symptom of scleroderma is harder and thicker skin in the facial regions such as the forehead and neck [19]. Scleroderma can happen to up to 50% of diabetic patients. MRI and CT scan images are used for analysing facial morphological changes. Scleroderma is diagnosed through imaging photoplethysmography [34].

The objective of this study is to analyse a person with DM through the vision diabetology method. The symptoms of dermatological issues are extracted and classified using the MWTCNN algorithm. The MWTCNN model is compared with other deep learning models such as VGG19, Inception V3, and ResNet-50.

## 2. Methodology

### 2.1. Material Preparation

Numerous skin conditions on the face focus on examining diabetes through the face region. According to the American Diabetes Association, diabetes destroys small blood vessels, leading to various skin conditions on the entire body. The face is a noticeable area, making skin damage simple to spot. The several skin conditions that affect the face include Bell's palsy [29], vitiligo [21], rubeosis faciei [17], and scleroderma [33]. Patients with a high glycemic index, those who have had diabetes for more than ten years, and those who are in the early stages of the disease all experience blood vessel damage [35].

### 2.2. Skin-Related Data Collecting on the Face

The patient's family history of diabetes, average age of over 54 years, and presence of diabetes in both sexes were evaluated. Data were gathered in the winter when sunshine exposure was minimal. The patient's video has been gathered. Based on the solar region, the patients were separated into two groups. Patients with a white complexion and an erratic fasting hyperglycaemic index can be easily recognized as having rubeosis faciei. Bell's palsy happens when facial movement is lost. Bell's palsy makes it challenging to frown, smile, or make other facial movements. Bell's palsy can develop quickly; symptoms and paralysis can manifest as early as 48 hours after the condition first manifests. The prognosis for Bell's palsy patients is generally favourable; about 90% of them recover fully. In addition, this harms tiny blood vessels. The patient's mild and full smiles are used to collect Bell's palsy patient videos.

Diabetes patients with types 1 and 2 are likelier to get vitiligo skin conditions [21]. Vitiligo happens as a result of the destruction of specific skin-colouring cells [21]. Vitiligo can happen everywhere on the body, but the mouth, nose, and eyes are the most common places to find it [36]. Compared to persons with a white complexion, those with dark skin can be easily identified. Thus, data from videos of persons with dark complexions is gathered. Face, finger, and hand skin become thinner due to scleroderma. It is brought on by a type 1 diabetic patient's long-term diabetes. It results from a slight nerve injury. The patient's face's skin is hardening because they have had diabetes for over 15 years. The facial skin region of the patient with type 1 diabetes is employed in the video collection. It happens to both those with light and dark skin.

**Figure 2:**
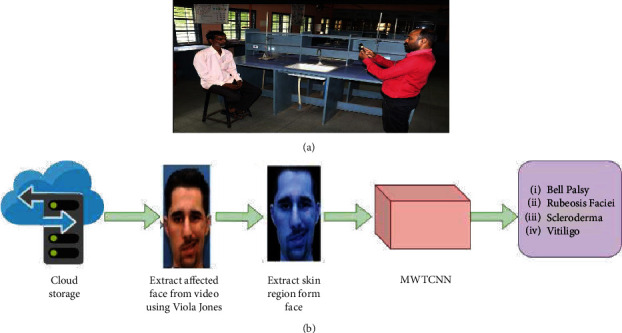
(a) Experimental setup. (b) Block diagram of vision diabetology-MWTCNN facial skin disease.

Figure 2(a) shows the experimental setup. We employ an automated method to detect diabetes in the skin region of the face. A mobile-based IP web camera is mounted on a tripod at a height of 75 cm. A person was sitting on the chair, and the distance between the tripod and the person was 3 cm. The camera is angled at 90 degrees, has a resolution of 1600 × 720 pixels, is front-focused, and shoots at 30 frames per second.

Compared to the Canon 360ES camera, the IP webcam is portable, free, and produces high-quality video. The videos are saved immediately to the supplied email addresses on Google Drive. We can readily access the data whenever we want, which is more secure. We considered a consistent background, a stable camera, nonmoving objects with minimal reflections and shadows, a short distance, and the right angle and viewpoint.  Figure 2(b) explains the steps followed in vision diabetology.

**Figure 3:**
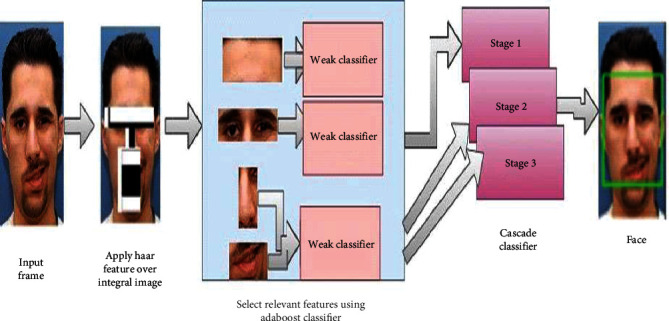
Viola Jones algorithm.

Viola Jones' face detection method is used to analyse the video clip [6].  Figure 3 shows the steps to detect the affected face from the video. Using Haar's features, the system recognizes the face automatically. The algorithm detects eyes, nose, and mouth using Haar features. The Haar features are computed between lighter and darker pixels around the eyes, mouth, and nose. The HSV colour segmentation algorithm is used to determine the skin region of the face. The extracted skin regions are passed through an MWTCNN network to classify different skin conditions of the facial region and analyse persons' diabetes.

### 2.3. Skin Extraction from Face Region

Skin extraction from the face region is an important step in diagnosing diabetes **in** diabetic patients. The HSV colour model is used to segment the skin region of a diabetic person. The human skin tone can vary due to the varying colour spectrum in the skin as well as lighting, illumination, contrast, brightness, and saturation. The skin area is separated from the facial image by considering the pixels in the face area. Hue, saturation, and value are the three components of the HSV colour model. The major colour viewed by humans is hue. The saturation represents the amount of white light with the hue, and the value denotes the pixel's brightness. An HSV colour space can be visualized as a geometric cylinder, with the angular dimension representing hue (H), beginning with primary red at 0°, going to primary green at 120°, primary blue at 240°, and returning to red at 360°. Saturation corresponds to the distance from the centre axis of the HSV cylinder (S). A saturation number rising towards the outer edge indicates that the colourfulness is increasing. The **v**alue (**V**) axis is the central vertical axis of HSV colour space, running from black at the bottom with lightness or value 0 to white at the top with a value of 1.

**Figure 4:**
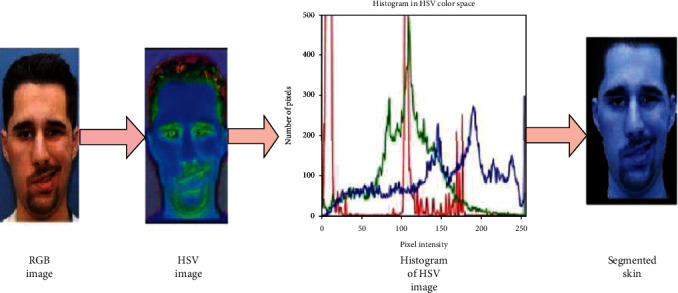
Result obtained from the HTHSV model for Bell's palsy.

Human skin colour is distributed based on the equator and temperate regions. Human skin tone variation and solar radiation variation by location are closely connected. Human skin colour used to have a gradual distribution until human migration grew considerably during the last several hundred years. Dark skin was more concentrated around the equator, where solar radiation was strong, while lighter skin was found farther north or south of the equator as solar radiation strength decreased. So we have proposed a hue-based threshold HSV (HTHSV) colour model for the skin region of the human face. The HTHSV algorithm is provided in Algorithm 1. We set the saturation value based on skin tone and solar radiation. Saturation is defined as the ratio of white light to hue. In our experiment, we assigned a value ranging from 60 to 150, depending on the equator and temperate zone. We assigned a hue value ranging from 120 to 179.  Figure 4 shows the result of the HTHSV model.

### 2.4. Multiwavelet Transform Convolution Neural Network

**Figure 5:**
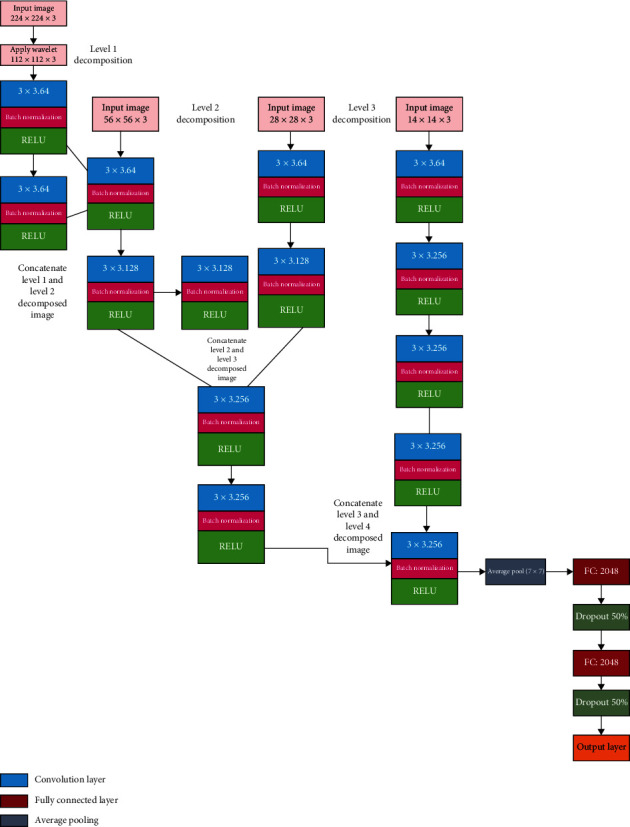
Architecture of vision diabetology-multiwavelet CNN (VDMWCNN).

**Figure 6:**
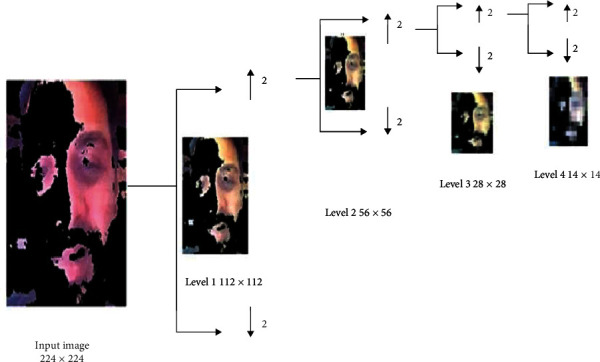
Convolution of RGB image.

The vision diabetology multiwavelet CNN (VDMWTCNN) can be seen in Figure 5. The wavelet transform considers both the time and frequency domains. Wavelet filters produced good results for low-resolution components compared to other filters. Here, the skin tone varies from person to person in various face skin region locations depending on the situation. Therefore, we must consider frequency domain data to distinguish between the affected and normal skin, as frequency domain features are utilised to measure brightness and detect changes in skin colour. Because a patient's skin alterations develop when they have had diabetes for more than ten years, the temporal domain reflects changes over a specific period. We have developed the MWTCNN network to address the issue mentioned above.  Figure 6 illustrates how our network translates the RGB colour image of size 224 × 224 into 4-level decompositions of images such as 112 × 112, 56 × 56, 28 × 28, and 14 × 14.

The extracted RGB colour image's skin area is separated into 4 distinct subband images. We have independently broken down the RGB image into the red, green, and blue components that are shown in equations (1), (2), and (3). Next, each component is subjected to a different wavelet decomposition process. As a result, we can obtain four subimages: a low-pass approximation image and three high-pass images, including horizontal, vertical, and diagonal images. The size of the decomposed image was decreased to half that of the original image during decomposition. We applied a second decomposition to the low-pass approximated image, creating 4 subimages. The same procedure is repeated up to four sublevels. Each RGB colour component of the image is processed separately. The components are finally combined. (224 × 224) is the RGB image's original size, which is divided into (112 × 112), (56 × 56), (28 × 28), and (14 × 14).

(1) $$\begin{array}{c} R=RGB\left(:,:,1\right), \end{array}$$

(2) $$\begin{array}{c} G=RGB\left(:,:,2\right), \end{array}$$

(3) $$\begin{array}{c} B=RGB\left(:,:,3\right), \end{array}$$

(4) $$\begin{array}{c} R_{i}=conv\left(R,f_{i}\right). \end{array}$$

Equation (4) *R*_*i*_ is indicated as the feature map of the red component and is referred to as the convolution of the red component (*R*) with the *f*_*i*_ Haar filter, “*i*” gives the details of feature maps such as approximation, vertical, horizontal, and diagonal images. Equations (5) and (6) represents the convolution operation of the green and blue component.

(5) $$\begin{array}{c} G_{i}=\text{conv}\left(G,f_{i}\right), \end{array}$$

(6) $$\begin{array}{c} B_{i}=\text{conv}\left(B,f_{i}\right). \end{array}$$

The orthogonality of wavelet reveals twin-scale relation, that is, decomposition and reconstruction due to orthogonal wavelet relation. Because there is no information loss due to orthogonality, we can reconstruct the image, and the lossless information gives the correct identification of DM through the skin region, so we presented a wavelet-based multiresolution convolution network for the facial skin region.

### 2.5. Network Architecture of MWTCNN

Figure 5 depicts a multiwavelet convolution neural network to classify the various DM face skin disorders. The proposed MWTCNN model captures complex features, skips connections to avoid the vanishing gradient problem, and improves the training process. The model captures multiscale features compared to CNN models like VGG 19, ResNet-50, and Inception V3. The proposed model utilizes both spatial and frequency information and improves gradient flow. We utilised a convolution neural network with 15 layers without any pooling. Each convolution layer comprises three blocks: a convolution block, a batch normalizing layer, and a rectified linear unit. Each convolution layer uses a distinct 3 × 3 kernel filter with 1 × 1 padding to evaluate both low-pass and high-pass images as input. Skin tone varies from person to person and from face to face due to temperature, lighting, brightness, and contrast to obtain more precise information on the feature map. So, a single image is separated into four subbands, and each subband level is handed to the convolution layer, which has 64,128,256 filters.

In this case, we used both upsampled and downsampled photos to ensure that no information was lost in the given input image. Because more detailed abstract information is derived from images, the number of filters should be high. Initially, the convolution network employs raw pixel information in an image. In order to prevent it, we first built up 64 filters with 3 × 3 pixels so that each neuron could think of its upper, lower, left, and right neighbourhoods as having a total of eight neighbourhoods. The batch normalization layer optimizes the input, making training simple and quick. If given a negative input, the rectified linear unit returns zero; otherwise, it gives the same value.

As a result, each subband was convolved with two sets of three 3 × 3 convolution layers. The level 1 decomposed input of 112 × 112 was then blended with the next three convolution layers with the level 2 decomposed picture of 56 × 56 inputs with filters of 64 and 128. The level 1 and 2 images are blended with the input image of size 28, 28, and 3 × 3 convolution kernels with 64,128 and 256 filter sizes. The level 1, level 2, and level 3 subimages are then combined using a 3 × 3 convolution neural network with 64,256 filters. Because conventional CNN's pooling layer only examines maximum values, we experience higher information loss. We removed max pooling by adding a batch normalization layer to avoid the issue above.

Finally, average pooling with a 7 × 7 kernel and a stride 1 × 1 is introduced. The feature vector of 2046 was then added to two fully connected layers. Finally, the dropout layer removes 50% of the feature vectors to speed up training and classify the output.

#### 2.5.1. Model Details

We have created a multiwavelet convolution neural network based on vision to categorise various skin conditions and detect diabetes in the face region. The network is built using the Keras toolkit and TensorFlow. At the network's finish, a dense classifier for the facial skin region is added. The model information is displayed in Table 1. The optimizer is Adam optimizer, and the environment is Google Colab. The loss is sparse categorical loss, the learning rate is 0.001, and there are 10 epochs. The batch size for training was 8, and the dropout rate was 0.5%. The model was trained in the Google Colab TPU environment.

## 3. Results

Table 2 displays each model's classification accuracy and running time in various face skin regions. The findings show that the four models categorise facial skin dermatological issues. The VGG 19 model, ResNet-50 model, Inception V3 model, and our model all produce high classification accuracy ratings of 88.3%, 86.6%, 88.3%, and 98.3%, respectively. The rationale for our suggested model's maximum classification accuracy is that we used up and downsampled images. Therefore, no information is lost. The training time of the models is short due to the small dataset. The training time for the models is 32 s, 35 s, 40 s, and 30 s. Our dataset contains 200 images. The entire dataset is divided into 70 and 30% of data. The evaluation metrics are shown in the following equations:

(7) $$\begin{array}{c} \text{True positive}\left(\text{TP}\right)=\text{The model correctly identifies skin disease},\\ \text{True negative}\left(\text{TN}\right)=\text{The model correctly identifies patients skin as healthy},\\ \text{False positive}\left(\text{FP}\right)=\text{The model incorrectly identifies patients healthy skin affected with disease},\\ \text{False negative}\left(\text{FN}\right)=\text{The model fails to detect the skin disease},\\ \text{Precision}=\frac{\text{TP}}{\left(\text{TP}+\text{FP}\right)},\\ \text{Recall}=\frac{\text{TP}}{\left(\text{TP}+\text{FN}\right)},\\ F1-\text{score}=\frac{2\times \left(\text{precision}\times \text{recall}\right)}{\left(\text{precision}+\text{recall}\right)},\\ \text{Accuracy}=\frac{\text{TN}+\text{TP}}{\text{TN}+\text{TP}+\text{FN}+\text{FP}}. \end{array}$$

**Figure 7:**
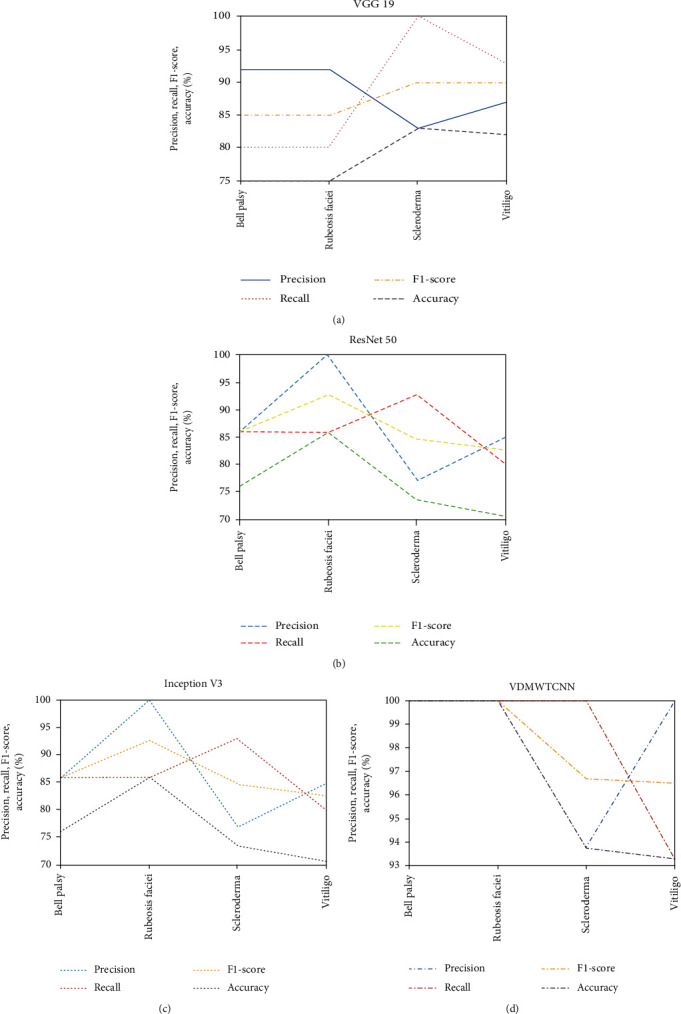
Precision, recall, *F*1-score, and accuracy of (a) VGG 19, (b) ResNet-50, (c) Inception V3, and (d) VDMWTCNN.

Figure 7 shows the precision, recall, *F*1-score, and accuracy results from our suggested model and VGG19, ResNet-50, and Inception V3. Our suggested model produces high precision, recall, *F*1-score, and accuracy in DM dermatological issues compared to previous CNN models. The wavelet analyzer works like a microscope, focusing on the smallest aspects of blood vessel damage. Therefore, the accuracy of diseases is automatically increased by our suggested model.

**Figure 8:**
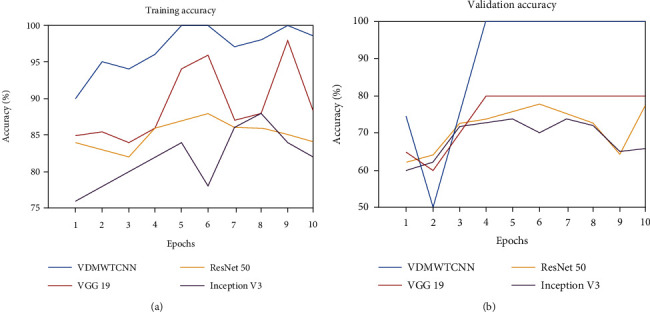
(a) Training accuracy curve. (b) Validation accuracy curve.

Figure 8 shows that the validation accuracy of the MWTCNN model is greater than the training accuracy. As a result, our model fits the data perfectly. However, the validation accuracy of other models, like VGG 19, ResNet-50, and Inception V3, could be higher than the training accuracy, indicating that all models overfit the data. Therefore, we must increase the sample size and train the model over more epochs. Our data was divided into two sets, with 30% of the data utilised for the test set and 70% of the data used for training. The performance of our network has been enhanced by adding two dropout layers, and the model now trains more quickly with 10 epochs.

**Figure 9:**
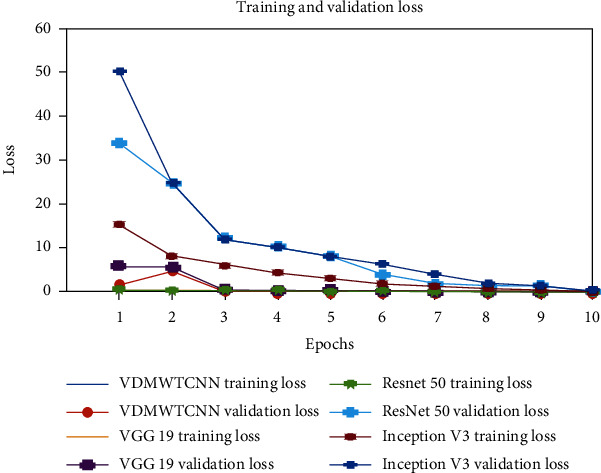
Training and validation loss.

The validation loss is used to fit new data into the model, and the training loss illustrates how well the model fits the data. The model is said to be underfitted to the data when the loss function is large and the loss value does not decrease over time. However, Figure 9 demonstrates that the training and validation losses are consistently decreasing, indicating that the models are effectively fitted to categorise the facial region of skin disorders and identify the patient's diabetes with ease.

**Figure 10:**
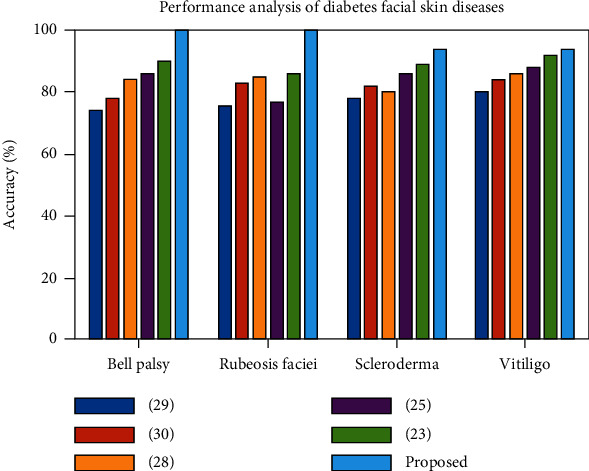
Overall performance of diabetes facial skin region.

The overall accuracy of our suggested model is shown in Figure 10. In comparison to [18, 25, 30, 31] and [29], our model produces the highest accuracy. In Bell's palsy, the facial nerve passes through a narrow bone corridor that paralyzes the facial region. In rubeosis faciei, the superficial venous plexus, such as a network of nerves located in the face, is dilated due to increased blood flow, which leads to redness in the face. Scleroderma damages the tiny blood vessels near the skin's surface, reduces facial movement, and decreases mouth opening. Vitiligo occurs as depigmentation of the skin due to increasing blood flow. Random matrix theory (RM) [25] does not apply to all skin pattern changes, such as a network of nerves, the nerves in the corridor of bone and increasing blood flow in a particular area of nerve because of its complex analysis, such as eigenvalues, eigenvectors, and spectral analysis. The assumptions of statistical properties for analyzing random matrices are independent of matrix elements. Hence, analyzing skin patterns using a random matrix may vary depending on the nature of skin dryness and colour variations. RM led to inaccurate results for facial dermatological issues. Temporal facial colour (TFC) [18] variation videos are captured under noncontrollable lighting conditions. Lighting conditions affect the appearance of rubeosis faciei due to the vascular dilation of the superficial venous plexus, and lighting conditions affect the blood flow changes in the affected area. TFC causes inaccurate facial colour changes and issues in showing accurate small blood vessel damage.

Hence, the above method produces less accuracy for all facial dermatological issues. The laser speckle contrast imaging (LSCI) [30] technique is used to visualize the blood flow in facial nerves. However, LSCI leads to errors in detecting dermatological changes in facial skin because of speckle size, speed of motion, and slow blood flow movement. LSCI can monitor blood flow changes in vitiligo, microvascular function in scleroderma, and blood flow changes in the superficial nerve of the venous plexus. However, Bell's palsy occurs due to swelling of the facial nerve. Hence, LSCI is not applicable to Bell's palsy. The drawbacks mentioned above are avoided using the deep learning model because MWTCNN directly works on pixels, utilizes both upscaled and downscaled information in images, and captures local and global features of skin lesions. Hence, our proposed model produces a high accuracy of 98.3% compared to other models.  Table 3 shows the accuracy of facial dermatological issues in different models.

**Figure 11:**
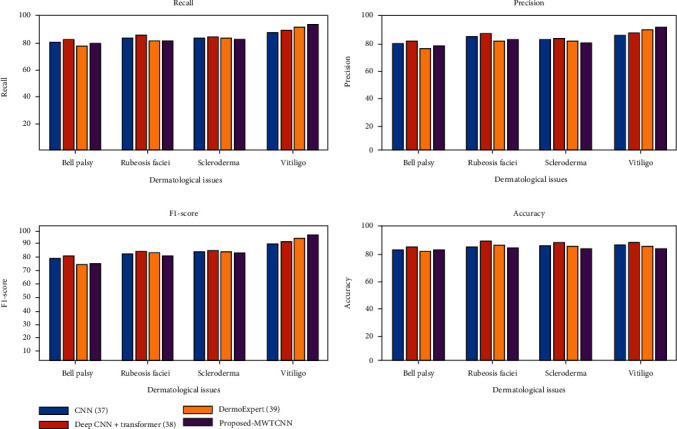
Precision, recall, *F*1-score, and accuracy of proposed and other CNN models.

Figure 11 shows the precision, recall, *F*1-score, and accuracy of the proposed and other CNN models. The convolutional neural network model [37] needs a large amount of data to classify facial skin dermatological issues. The CNN model overfits the data because we used only a small number of samples. The CNN model extracts features only from high-quality images. The model is applied to facial dermatological issues; it does not read the blood flow rate accurately. The blood flow changes the facial features sometimes; the facial skin may be dry, bright, and shiny. The CNN model cannot extract features with dryness, brightness, and shining images. The deep CNN with transformer model [38] uses all parts of the input image by dividing the input image into tokens and applying the transformers directly to the sequence of input images. The deep CNN with transformer model does not highlight the localized information, is unable to capture the contextual information, and is applicable for large datasets. In our dataset, we focused on small blood vessel damage, and it was not captured accurately by the deep CNN transformer model. Hence, the model produces low accuracy, precision, recall, and *F*1-score values. The DermoExpert [39] model contains preprocessing, segmentation, and classification. The DermoExpert model uses sequences of input images with three-level feature maps. The feature maps downsample the input image and decrease the vanishing gradient problem. The model used only downsampled images. Hence, the model needs to produce more accuracy. The proposed MWTCNN model uses spatial and spectral features and multiscale features, and the model utilizes both upscaled and downscaled information. Hence, there is no information loss. So, our model produces 98.3% classification accuracy.

## 4. Discussions

The MWTCNN model was developed to assess DM severity via the skin of the face. Skin lesions on the face are linked to DM in this model. Damage to these capillaries in the face due to DM leads to dermatological issues. It has been found through a review of the relevant literature that many deep learning models have been developed to analyse the skin lesions associated with DM based on facial skin conditions such as Bell's palsy, rubeosis faciei, scleroderma, and vitiligo. However, they do not have a causal relationship with patients' glucose levels. Fasting blood glucose, random blood glucose, oral glucose tolerance test, and haemoglobin A1c test are some examples of blood glucose measurements. There are drawbacks to diabetic measures, such as higher costs, less precise measurements, and a lack of standardisation of blood glucose levels. As a result, our proposed deep-learning model examines a specific area of the patient's face and calculates the prevalence of diabetes in record time for continuous glucose monitoring. Using both upscaled and downscaled images, with the network preserving information and expanding the receptive field, is just one of the many benefits of the proposed MWTCNN model. Compared to other networks, such as VGG19, Inception V3, and the ResNet-50 model, the proposed model achieves superior accuracy, precision, and recall. The VGG19 needs more time to train the images because of the greater number of parameters. The Inception V3 model employs a maximum pooling operation, which increases the model's computational complexity and lengthens the time required for training to determine the optimal weight. As a result, knowledge about skin lesions on the face has yet to be considered. More memory is required for the ResNet-50 model. As a result, the proposed model outperforms in terms of accuracy, sensitivity, precision, and recall.

### 4.1. Limitations

Even though the proposed MWTCNN model is trained using a small number of images, it uses the spectral and spatial information of facial skin lesions. In addition, the model is enhanced using regularisation techniques to prevent overfitting and enhance precision and training speed.

## 5. Conclusion

Many people, young and elderly, are affected by DM. DM affects tiny blood arteries. The facial skin arteries are visible. The skin area of the human face allows us to identify diabetic patients quickly. We used a video-based vision diabetology MWTCNN model to detect and categorise facial skin dermatological issues, and we got the maximum recognition accuracy of 98.3%. Deep learning algorithms are used in the proposed vision diabetology method to analyse the early signs of diabetes in the area of the facial skin. The suggested methodology makes use of low-risk, economical techniques. A small number of diabetic persons are involved in the preliminary study. Future studies will include the amount of infection and retinopathy, prediabetic, family history of diabetes, and diabetic levels.

## Figures and Tables

**Algorithm 1:**
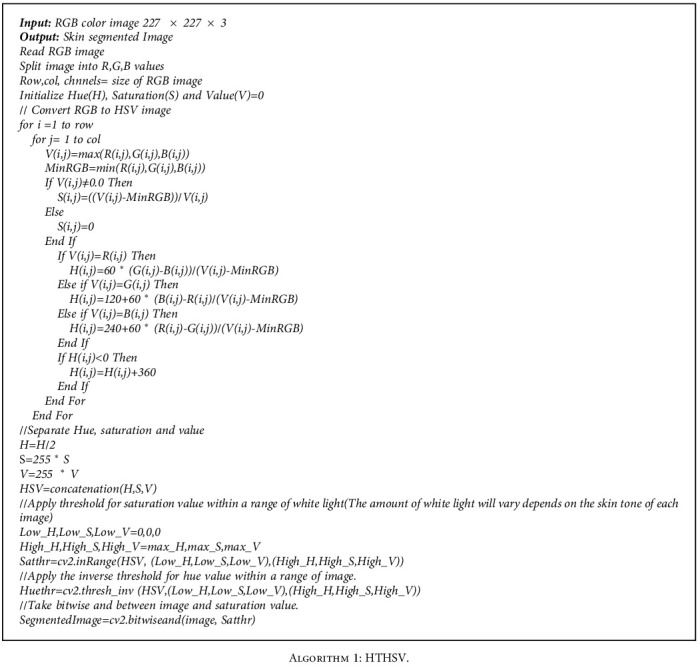
HTHSV.

**Table 1:** Model implementation.

Input	224 × 224 × 3
Number of layers	59
Classes	4
Total parameters	21,494,340
Optimizer	Adam
Learning rate	0.001
Batch size	8
Number of epochs	10
Dropout	0.5
Environment	TPU/Google Colab
Platform	Python 3.6

**Table 2:** Overall classification accuracy of VGG19, ResNet-50, and Inception V3.

Model	Classification accuracy	Running time
VGG 19 [29]	88.3%	32 s
ResNet-50 [30]	86.6%	35 s
InceptionV3 [31]	88.3%	40 s
WTCNN (proposed)	98.3%	30 s

**Table 3:** Comparison with other models.

S. no	Model	Face region	Accuracy (%)
1	[18]	Temporal facial region	Bell palsy—78%
			Rubeosis faciei—75%
			Scleroderma—80%
			Vitiligo—79%

2	[25]	Random matrix theory	Bell palsy—79%
			Rubeosis faciei—83%
			Scleroderma—78%
			Vitiligo—85%

3	[30]	Laser speckle contrast imaging	Bell palsy—76%
			Rubeosis faciei—82%
			Scleroderma—84%
			Vitiligo—86%

4	[31]	Temperature and texture features	Bell palsy—78%
			Rubeosis faciei—84%
			Scleroderma—80%
			Vitiligo—84%

5	Proposed	MWTCNN	Bell palsy—99%
			Rubeosis faciei—99%
			Scleroderma—98%
			Vitiligo—98%

## Data Availability

The datasets used and/or analysed during the current study are available from the corresponding author upon request.
